# Association Between Cervical Lordosis Restoration and Clinical Recovery Following a Motor Vehicle Collision: A Chiropractic BioPhysics Case Report With a 17-Month Follow-Up

**DOI:** 10.7759/cureus.115219

**Published:** 2026-08-26

**Authors:** Thomas J Woodham, Paul A Oakley, Miles O Fortner, Jason W Haas, Deed E Harrison

**Affiliations:** 1 Chiropractic, Western Plains Chiropractic, Gillette, USA; 2 Chiropractic, Private Practice, Newmarket, CAN; 3 Research, CBP (Chiropractic BioPhysics) NonProfit, Inc., Windsor, USA; 4 Research, CBP (Chiropractic BioPhysics) NonProfit, Inc., Eagle, USA

**Keywords:** cervical kyphosis, cervical lordosis, headache, motor vehicle collision, neck pain, traction, whiplash, whiplash-associated disorder

## Abstract

We describe the clinical and radiographic outcomes of a patient presenting with post‑traumatic loss of cervical lordosis following a motor vehicle collision (MVC). A 27‑year‑old man reported to a spine rehabilitation clinic complaining of neck pain, low back pain, and headaches following an MVC one month prior. The patient scored moderate disability: 36% and 34% on the neck and headache indices, respectively. Radiograph screening of the cervical spine demonstrated severe anterior head translation (AHT) of 59.9 mm (ideal = 0 mm), complete loss of the cervical lordosis (C2-C7 absolute rotation angle = -1.6°; ideal = -42°), and a sharp reversal of the C5-C6 intersegmental angle of +5.9° (ideal = -8.0°). Chiropractic BioPhysics® (CBP®) (Chiropractic BioPhysics Seminars, Eagle, ID, USA) treatment aimed at restoring cervical lordosis and sagittal balance was performed over 6.5 months with a 17-month follow-up. Symptomatic improvement progressed to being negligible over the 6.5-month treatment duration. At the final follow-up, radiographic parameters improved by 38 mm in AHT, 17° in lordosis, and 10.5° in the C5-C6 segmental kyphosis. This case illustrates that meaningful structural correction is achievable and may coincide with profound and sustained clinical recovery, supporting further investigation into sagittal alignment restoration as a central therapeutic objective in patients suffering from cranio-cervical symptoms following an MVC.

## Introduction

The cervical lordosis is a fundamental structural feature of the human spine that plays a critical role in load distribution, shock absorption, horizontal gaze, and overall sagittal balance [1-3]. A normal cervical curvature allows for the efficient transmission of axial loads through the vertebral bodies and posterior elements while minimizing stress on the intervertebral discs, ligaments, and musculature [2,4]. Loss or reversal of the cervical lordosis has been associated with increased mechanical strain, altered spinal biomechanics, accelerated degenerative changes, and a variety of clinical symptoms including neck pain, headaches, radiculopathy, and reduced quality of life [5-11]. Restoration of physiologic cervical alignment has therefore emerged as an important structural goal in spine rehabilitation [12-14].

Motor vehicle collisions (MVCs), particularly rear‑impact collisions, are well known to disrupt cervical spine alignment [15-19]. Acceleration-deceleration mechanisms can result in anterior head translation (AHT), segmental instability, muscular guarding, and straightening or kyphotic reversal of the normal cervical curve [20,21]. While conventional management often focuses on symptom control, emerging biomechanical evidence suggests that persistent sagittal misalignment may perpetuate abnormal loading patterns and contribute to chronic pain syndromes. Structural rehabilitation approaches that aim to restore cervical lordosis may therefore offer advantages beyond temporary symptom relief by addressing the underlying postural deformity, particularly for subjects involved in MVCs [22].

Chiropractic BioPhysics® (CBP®) (Chiropractic BioPhysics Seminars, Eagle, ID, USA) is a structural rehabilitation method that applies the Mirror Image® (Chiropractic Biophysics Seminars, Eagle, ID, USA) concept through corrective exercises, spinal adjustments, and extension traction to improve spine and postural alignment [23]. Multiple reports have documented the capacity of cervical extension traction (CET) to increase cervical lordosis and reduce AHT [12,14,21,22]. The purpose of this case report is to describe the clinical and radiographic outcomes of a 27‑year‑old man presenting with post‑traumatic loss of cervical lordosis following an MVC, who underwent a CBP®-based corrective program aimed specifically at restoring cervical curvature and sagittal balance.

## Case presentation

In October 2022, a 27-year-old man (height = 191 cm; weight = 118 kg) presented complaining of neck pain, low back pain (LBP), and headaches following an MVC one month prior. Following the collision, the patient was nauseated and lightheaded. He was hit from behind at approximately 40 mph. He saw the car coming and had his seatbelt on; however, the airbags did not deploy. Thirty minutes after the collision, he started to experience the onset of pain motivating him to attend a local hospital where he received a CT scan of the cervical spine and an X-ray of the lumbar spine. He was discharged and given the muscle relaxant cyclobenzaprine and hydrocodone-acetaminophen for pain control. The patient was rear-ended one year prior, as a passenger, but reported to be okay after that collision.

At the time of the exam, the patient reported experiencing an average pain intensity of 3/10, 1/10, and 3/10 for the cervical, thoracic, and lumbar areas, respectively (Table 1). The neck pain and LBP were described as constant, and the headaches were reported as daily. Since the collision, the patient also reported experiencing many other symptoms (Table 2), including pain in the right shoulder, anxiety, coldness in the legs after sitting for 30 minutes or longer, as well as brain fog and cognitive delays. He also reported diarrhea, lack of energy, ringing in the ears that was worsened, and trouble sleeping due to pain after the incident. The patient scored a 36% on the Neck Disability Index (moderate disability) [24], a 34% on the Headache Disability Index (moderate disability) [25], and a 32% on the Oswestry Low Back Pain Disability Index (moderate disability) [26] (Table 1). On the 36-Item Short Form Health Survey (SF-36) quality of life questionnaire, the patient scored low on many indices [27] (Table 3).

**Table 1:** Average pain intensity and disability reporting.

	Baseline	3.75 months	6.5 months	17 months
Average pain intensity				
Cervical	3/10	1/10	0/10	0/10
Thoracic	1/10	0/10	0/10	0/10
Lumbar	3/10	0/10	0/10	0/10
Disability index				
Neck Disability Index	36%	16%	2%	0%
Headache Disability Index	34%	0%	0%	6%
Oswestry Disability Index	32%	6%	2%	0%

**Table 2:** Patient symptoms with percentage improvement at 24 sessions, 48 sessions, and 17 months f/u. f/u: follow-up

Initial symptoms	After 24 sessions	After 48 sessions	17 months f/u
Neck pain	20% improved	90% improved	100% improved
Low back pain	80% improved	100% improved	100% improved
Headaches	20% improved	90% improved	90% improved
Lightheadedness	30% improved	90% improved	90% improved
Nausea	80% improved	100% improved	100% improved
Right shoulder pain	10% improved	70% improved	70% improved
Coldness in the thighs	20% improved	90% improved	100% improved
Diarrhea	10% improved	90% improved	100% improved
Lack of energy	40% improved	80% improved	60% improved
Shortness of breath	70% improved	90% improved	90% improved
Ringing in the ears	20% improved	30% improved	No change
Difficulty sleeping	30% improved	100% improved	100% improved
Brain fog	40% improved	90% improved	90% improved
Anxiety with driving	Worse	40% improved	100% improved

**Table 3:** SF-36 health indices. 100 = ideal; normal values are shown. f/u: follow-up; SF-36: 36-Item Short Form Health Survey

	Health perception	Physical functioning	Physical	Emotional	Social functioning	Mental health	Bodily pain	Energy/fatigue
Normal	72	84	81	81	83	75	75	61
Baseline	32	65	25	0	63	52	58	30
3.75 months	50	50	100	33	63	52	80	55
6.5 months	67	95	100	100	88	64	90	60
17 months f/u	15	25	25	67	50	36	100	35

Posture assessment revealed several postural distortions, including a right translation of the head (-TxH), an anterior translation of the head (+TzH), a forward flexion of the head (+RxH), and a left-rotated pelvis (+RyP). Static palpation revealed hypertonicity, point tenderness, and motion restriction at C3, C4, and T8 and L4, L5, and S1. There were muscle spasms and contractures felt along the cervical, thoracic, and lumbar paraspinal areas. There were many positive orthopedic tests, including all cervical compression tests, Soto-Hall's, bilaterally for Kemp's, straight leg raise test, Patrick's, Nachlas', and Hibb's. Cervical distraction relieved the neck pain. Muscle strength testing showed decreased strength for cervical flexion (4), right cervical rotation (4), and bilateral hip abduction (4+). All cervical range of motion was restricted and painful except for right lateral flexion and right rotation. All lumbar range of motion was painful (except right rotation) and showed restricted motion. The deep tendon reflexes were normal. Sensory testing displayed hypoesthesia at C3 on the right and at L4 and L5 bilaterally.

**Figure 1:**
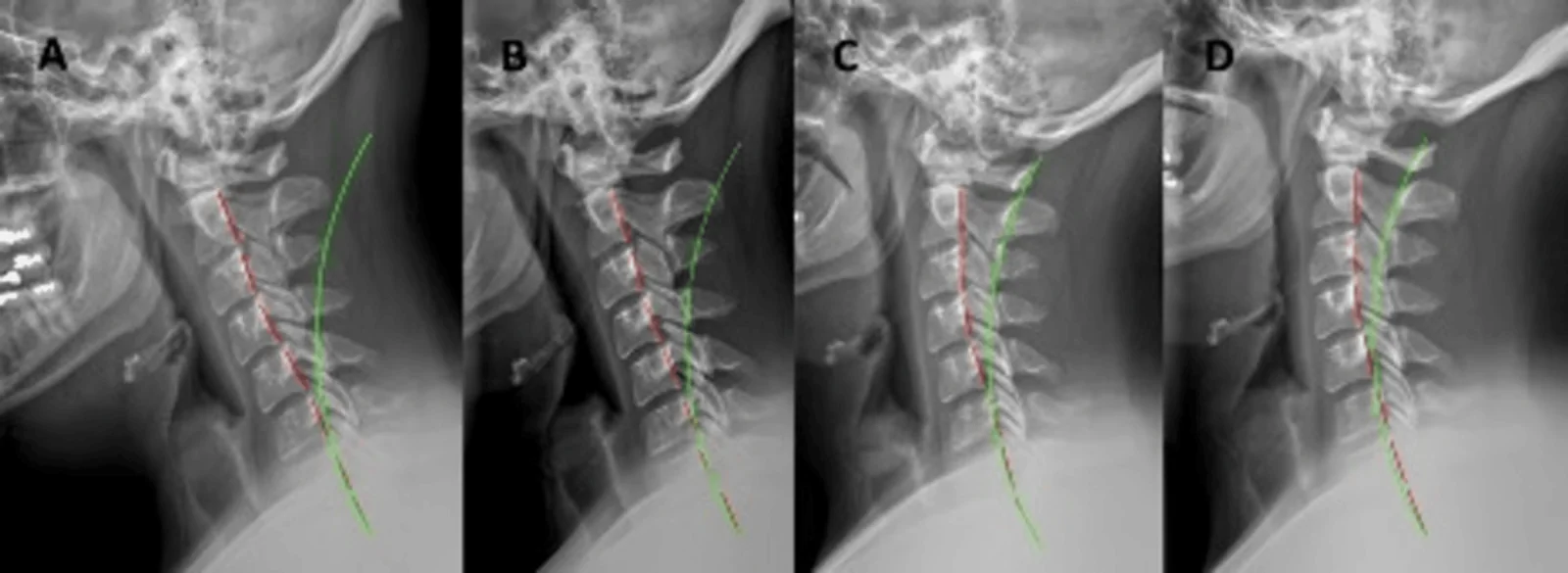
Lateral cervical radiographs: (A) pre-treatment, (B) first post-treatment, (C) second post-treatment, and (D) 17-month follow-up.

**Figure 2:**
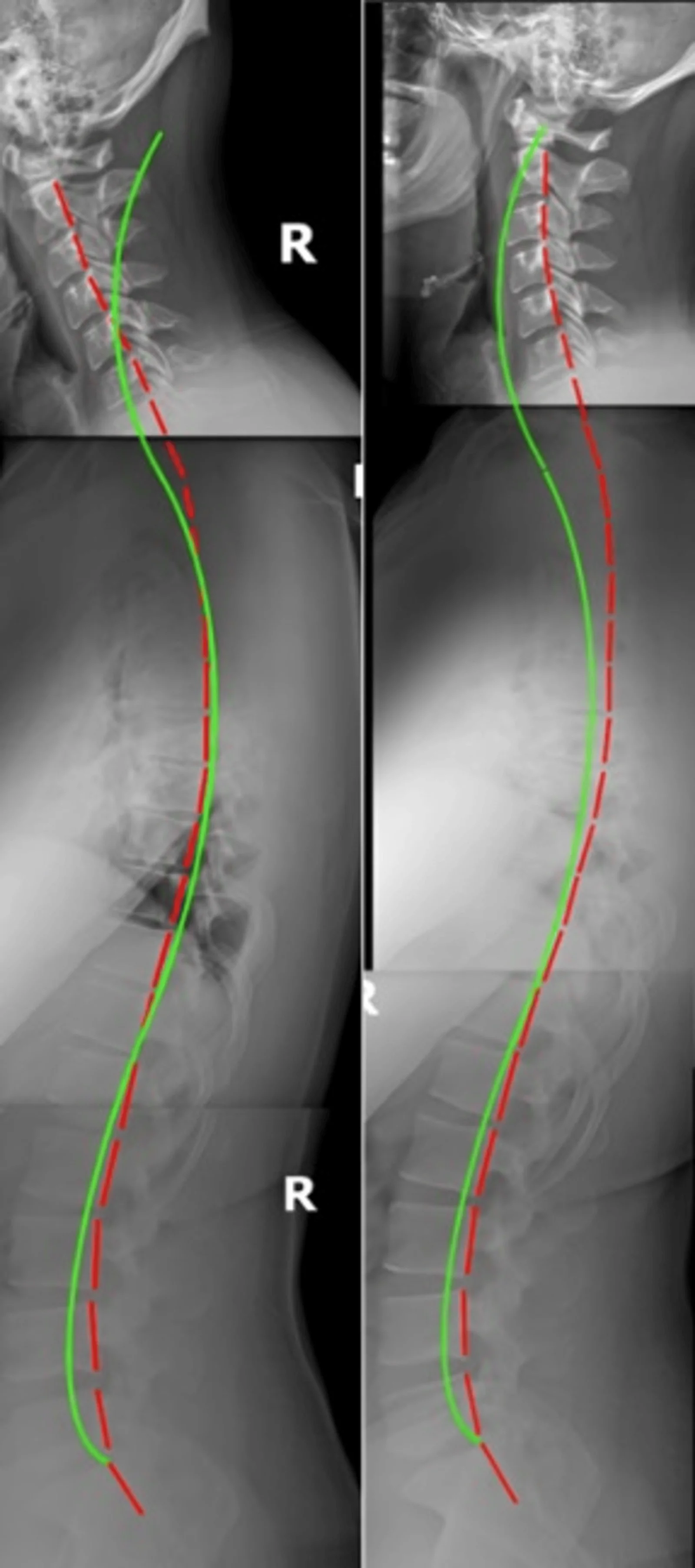
Lateral full spine radiographs: (left) pre-treatment and (right) follow-up.

Radiographic examination showed a forward hunched posture with the horizontal distance from C1 to S1 being +40.3 mm (Figure 1). There was severe AHT of 59.9 mm (ideal = 0 mm [28]) as well as complete loss of the cervical lordosis (C2-C7 absolute rotation angle (ARA) = -1.6°; ideal = -42° [28]) and a sharp reversal of the C5-C6 intersegmental angle of +5.9° (ideal = -8.0° [29]) (Table 4; Figure 2). The patient was in the neutral standing position for all X-rays. PostureRay® digitization software (PostureCo, Inc., Trinity, FL, USA) was used to measure all postural distortions [30]. Measurement error is within approximately 2° and 2 mm.

**Table 4:** Lateral cervical radiographic metrics. AHT: anterior head translation; ARA: absolute rotation angle; f/u: follow-up; m: month(s); RRA: relative rotation angle

Metric	Baseline	3.75 m	6.5 m	17 m f/u
Atlas plane line	-0.7°	-5.8°	-22.4°	-26.4°
C2-C7 ARA	-1.6°	-6.2°	-13.9°	-18.7°
AHT	59.9 mm	45.8 mm	25.6 mm	21.9 mm
C5-C6 RRA	+5.9°	+2.7°	+1.9°	-4.6°

**Figure 3:**
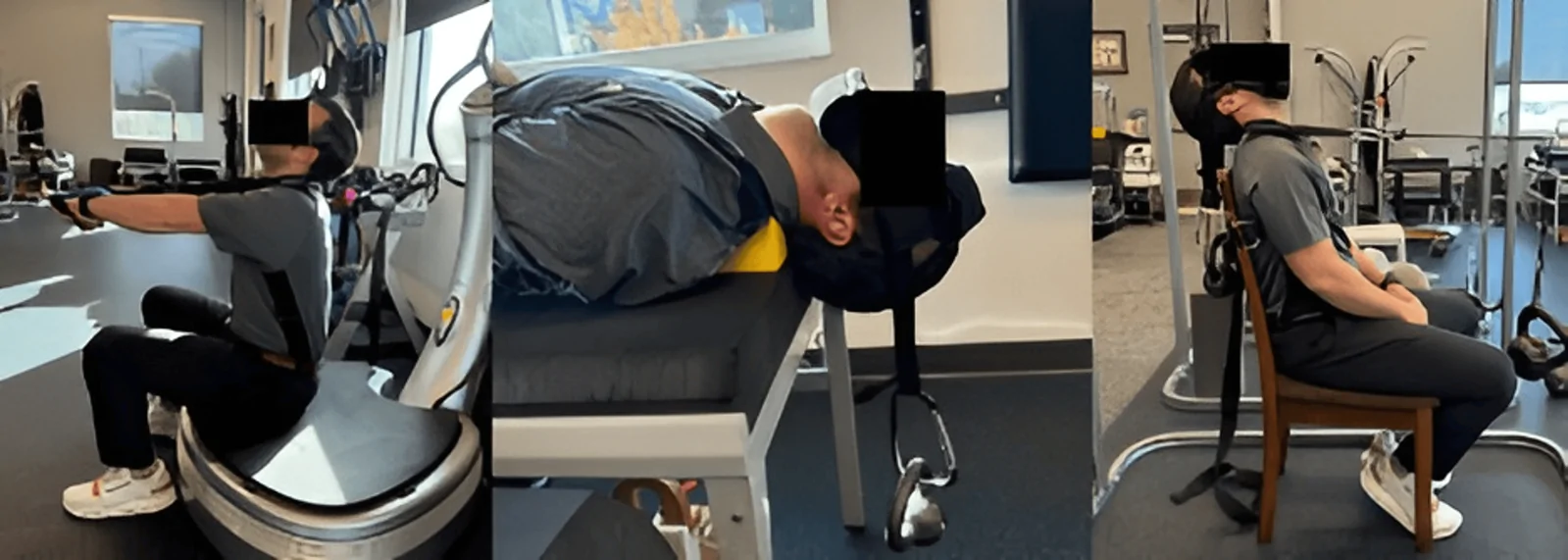
Left: cervical extension exercises on a Power Plate® vibration platform (Performance Health Systems, Northbrook, IL, USA). Middle: cervical extension traction with a small cervical Denneroll with a five-pound weight hanging from a chin-head harness. Right: seated Pope two-way traction with 20 pounds on the front and 10 pounds on the back. Traction was performed up to 15 minutes per session.

The current treatment protocol involved CBP® technique, and this case will describe the procedures used to restore the cervical lordosis [22,23,31]. CBP utilizes the Mirror Image® concept, such that cervical loss of lordosis is exercised, adjusted, and tractioned in a reverse manner in order to restore the normal lordosis. Corrective exercises involved cervical extension against resistance from the pro-lordotic exercise band (Circular Traction, Newport Beach, CA, USA). The strap was placed at mid-neck, and the handle was held with the thumbs pointing downwards. Each repetition was held for five seconds with a two-second rest in between reps and was performed while standing on the Power Plate® (Performance Health Systems, Northbrook, IL, USA) (Figure 3). Chiropractic adjustments consisted of both high-velocity, low-amplitude diversified manipulation to cavitate the facet joints and mirror image drop-table adjustments. CET was performed with a small cervical Denneroll with a five-pound weight hung with a chin-forehead strap which was performed for 15 minutes per session (Figure 3). Over time, as the patient easily tolerated this type of traction, they were graduated to a two-way CET with 20 pounds on the front and 10 pounds on the back, again performed for 15 minutes per session (Figure 3).

The patient was treated for 24 sessions over 3.75 months and then received another 24 sessions over 2.75 months, for a total of 48 sessions over 6.5 months. There was a follow-up assessment performed 17 months later. The patient consented to the presentation of these results.

Results

In February 2023, a reassessment was performed after 24 sessions. The patient reported improvement in all symptoms except anxiety with driving (Table 2). The LBP improved by 80%, while the neck pain and headaches improved by 20%. Static palpation revealed hypertonicity, point tenderness, and motion restriction at the occiput, C2, T7, and L5. Muscle spasms were experienced in the cervical and suboccipital region. Orthopedic compression tests were positive on the right, Soto-Hall's test was positive, Patrick's test was positive on the left, and Nachlas' and Hibb's tests were positive on the right. Muscle testing was positive for hip abduction as 4+ on the right. There was restricted motion in the cervical spine for flexion, extension, right lateral flexion, and left rotation, but without pain. Lumbar range of motion demonstrated no pain but restriction with flexion and right rotation and pain with restriction for extension and right lateral flexion. Sensory dermatome testing indicated hypoesthesia at L5 bilaterally. After 24 sessions, there were small improvements in AHT (45.8 mm vs. 59.9 mm), cervical lordosis (-6.2° vs. -1.6°), and the C5-C6 segmental relative rotation angle (RRA) (+2.7° vs. +5.9°). In March 2023, pain questionnaires were completed and demonstrated improvement in the Neck Disability Index (16% vs. 36%), Headache Disability Index (0% vs. 34%), and Oswestry Disability Index (6% vs. 32%), as well as 6/8 indices on the SF-36 (Table 3).

In April 2023, a reassessment was performed after 48 sessions. The patient reported 100% improvement in LBP and 90% improvement in neck pain and headaches. All other symptomatic complaints had improved as well (Table 2). Static palpation revealed hypertonicity, point tenderness, and motion restriction at C4, T9, and L3. All orthopedic tests, muscle strength testing, deep tendon reflexes, and sensory testing were normal. Cervical and lumbar range of motion were also normal, with the exception of restriction without pain during right lateral lumbar flexion. All pain indices were scored at 0-2% (Table 1), and there were further improvements in the SF-36 (Table 3). After 48 sessions, there was more improvement in AHT (25.6 mm vs. 45.8 mm), cervical lordosis (-13.9° vs. -6.2°), and the C5-C6 segmental RRA (+1.9° vs. +2.7°).

At the 17-month follow-up exam, the patient reported that all symptoms except the ringing in the ears had improved dramatically (Table 2). The posture was visually improved in terms of less AHT and no longer demonstrated a forward-flexed head position. Static palpation revealed hypertonicity, point tenderness, and motion restriction at C4, T7, and the sacrum. There were no muscle spasms or contractures experienced. All other tests were normal or within normal limits. The pain indices were scored at 0% except the HDI was 6% (Table 1) and some of the SF-36 scores had regressed (Table 3), although the pain index was completely alleviated (i.e., 100%). The long-term follow-up radiographic metrics (Figure 2) demonstrated dramatic overall improvement in AHT (21.9 mm vs. 59.9 mm), cervical lordosis (-18.7° vs. -1.6°), and the C5-C6 segmental RRA (-4.6° vs. +5.9°). The forward lean of the body had also improved (Figure 1).

## Discussion

This case documents the clinical and radiographic improvements following a structured CBP® program directed at restoring the cervical lordosis after a rear‑impact MVC. Initially, the patient presented with near-complete loss of cervical lordosis (C2-C7 ARA = -1.6°), marked AHT (59.9 mm), and a segmental kyphotic reversal at C5-C6 (+5.9°). Over 6.5 months and 48 treatment sessions, there was progressive structural correction, culminating at a 17-month follow‑up in a cervical lordosis of -18.7°, reduction of AHT to 21.9 mm, and normalization of the C5-C6 segmental angle (-4.6°). In this case, the patient was relatively young, which may enhance structural adaptability compared to older individuals; regardless, these structural changes paralleled the resolution of neck pain, headaches, and disability scores, suggesting a relationship between sagittal alignment correction and long‑term symptom resolution.

Whiplash-type injuries are known to alter cervical curvature and sagittal balance through rapid acceleration-deceleration forces that strain ligaments, discs, and facet capsules [16]. Persistent hypolordosis or kyphosis may increase anterior column loading, posterior element strain, and muscular demand required to maintain horizontal gaze [2-4]. Biomechanical modeling studies have demonstrated increased stress concentrations in non-lordotic cervical configurations compared to normal curvature [4], supporting the premise that structural deformity may perpetuate nociceptive input. In this case, initial symptomatic relief occurred alongside modest lordotic correction at 24 sessions, whereas complete resolution of disability and complex symptoms matched the substantial structural corrections achieved by 48 sessions, a trajectory consistent with reports where mechanical spinal realignment coincided with the resolution of multi-system complaints. This result is consistent with the recent report from Underhill et al. [22] who found modest improvements in cervical lordosis initially, but with continued treatment, clinical improvements mirrored structural improvements in the restoration of cervical lordosis in 51 patients.

The parallel improvements in clinical signs and symptoms and the structural improvements in cervical lordosis provide a temporal association that strengthens the hypothesis that restoring lordosis may contribute to durable recovery rather than transient symptomatic relief. At 17 months following the completion of care, improvements in cervical curvature and AHT were not only maintained but further improved. Pain and disability indices remained at or near 0%, with only minimal residual headache disability. In fact, the pain improvement of -3/10 was reduced beyond the minimal clinically important difference (MCID) of 1.5/10 points [32], and the Neck Disability Index also reduced (-36%) beyond the MCID of 15% [33]. The stability of radiographic correction, consistent with some long-term clinical trial data [14], suggests that CET and corrective exercises may induce viscoelastic and plastic deformation of anterior spinal structures, facilitating more permanent remodeling of sagittal alignment [34]. Long-term follow-up data remain limited in the whiplash literature; therefore, sustained improvements observed in this case add meaningful clinical insight.

Although this case supports the potential importance of cervical lordosis restoration, causation cannot be definitively established. Multimodal care was delivered, including spinal adjustments, exercises, and traction, and natural recovery cannot be fully excluded. However, spontaneous restoration of nearly 17° of cervical lordosis and correction of segmental kyphosis over time would be unlikely without targeted structural intervention. Furthermore, prior reports and controlled trials have demonstrated that CET is specifically associated with lordosis improvement [14,22], whereas conventional physiotherapy or manipulation alone typically does not yield comparable structural changes [35]. For example, Coleman et al. [36] demonstrate that activator adjustments resulted in increased cervical lordosis (6.4°); however, the improvements were small and likely resulted from the recovery of the strained musculature or due to head repositioning error [37]. In contrast, in this case, after one month's time, any immediate muscle strain could have subsided. Further, the 17° correction is substantially beyond head positioning error, supporting the rationale that the traction resulted in the lordosis correction. Importantly, while this case does not prove that lordosis restoration is the sole determinant of recovery, it supports the concept that structural correction may be a key component in successful management following MVC; this points to a unique structural approach that differs from traditional manual therapy approaches that aim to improve function only [38].

Several limitations must be acknowledged. This is a single case report without a control comparison, limiting generalizability. Although the whole spine of the patient was treated, this report focused on the symptomatic and structural changes in the cervical spine. It is important, however, to mention that improvements in non-cervical areas such as LBP also occurred; in fact, many published cases have reported full-spine improvements or regional interdependence of the spine [39] occurring such as the reduction in the forward lean of the body substantially improving in this case. Future prospective controlled trials with long-term follow-up are needed to clarify the degree to which restoration of cervical lordosis independently predicts improved outcomes after MVC. Nonetheless, this case illustrates that meaningful structural correction is achievable and may coincide with profound and sustained clinical recovery [22], supporting further investigation into sagittal alignment restoration as it may represent one potential contributor to recovery.

## Conclusions

This case demonstrates that targeted restoration of cervical lordosis and sagittal balance following MVC was associated with substantial and durable clinical improvement at the 17-month follow-up. Progressive correction of AHT, global cervical curvature, and segmental kyphosis paralleled the resolution of pain, disability, and functional limitations, suggesting that structural rehabilitation may play an important role in long-term recovery after whiplash-type injury. While causation cannot be established from a single case, the magnitude and persistence of radiographic and symptomatic changes support the premise that addressing post-traumatic sagittal deformity may represent a key therapeutic objective rather than focusing solely on short-term symptom relief. Further prospective, controlled investigations are warranted to determine whether restoration of cervical lordosis independently predicts improved outcomes and should be routinely incorporated into evidence-informed management strategies for patients following MVC.
